# Activated fibroblasts modify keratinocyte stem niche through TET1 and IL-6 to promote their rapid transformation in a mouse model of prenatal arsenic exposure

**DOI:** 10.1038/s41598-024-56547-8

**Published:** 2024-03-22

**Authors:** Anchal Chauhan, Siddhartha Gangopadhyay, Kavita Koshta, Sukhveer Singh, Dhirendra Singh, Vikas Srivastava

**Affiliations:** 1 Academy of Scientific and Innovative Research, Ghaziabad, 201002 India; 2 Systems Toxicology Group, FEST Division, CSIR-Indian Institute of Toxicology Research, Vishvigyan Bhawan, 31, Mahatma Gandhi Marg, Lucknow, 226001 Uttar Pradesh India; 3 Animal Facility, CSIR-Indian Institute of Toxicology Research (CSIR-IITR), Vishvigyan Bhawan, 31, Mahatma Gandhi Marg, Lucknow, 226001 Uttar Pradesh India

**Keywords:** Inorganic as, Prenatal exposure, Fibroblast activation, IL-6, EMT, Stem cell niche, Skin stem cells, Developmental biology, Diseases, Cancer microenvironment, Cancer stem cells, Skin cancer, Cell migration, Cell signalling, Cellular imaging, Mechanisms of disease

## Abstract

Early life exposure to environmental pollutants such as arsenic (As) can increase the risk of cancers in the offspring. In an earlier study, we showed that only prenatal As exposure significantly increases epidermal stem cell proliferation and accelerates skin tumorigenesis in BALB/c mouse offspring. In the present work, we have examined the role of As-conditioned dermal fibroblasts (DFs) in creating pro-tumorigenic niches for Keratinocyte stem cells (KSCs) in the offspring. DFs isolated from prenatally exposed animals showed increased levels of activation markers (α-SMA, Fibronectin, Collagen IV), induction of ten-eleven translocation methylcytosine dioxygenase 1(TET1), and secreted high levels of niche modifying IL-6. This led to enhanced proliferation, migration, and survival of KSCs. Increased IL-6 production in As-conditioned fibroblast was driven through TET1 mediated 5-mC to 5-hmC conversion at -698/-526 and -856/-679 region on its promoter. IL-6 further acted through downstream activation of JAK2-STAT3 signaling, promoting epithelial-to-mesenchymal transition (EMT) in KSCs. Inhibition of pSTAT3 induced by IL-6 reduced the EMT process in KSCs resulting in a significant decrease in their proliferation, migration, and colony formation. Our results indicate that IL-6 produced by prenatally conditioned fibroblasts plays a major role in regulating the KSC niche and promoting skin tumor development in As-exposed offspring.

## Introduction

The Developmental Origins of Health and Disease (DOHaD) hypothesis proposes that an individual’s early life experiences can strongly influence their health and susceptibility to disease in the future^1^. This theory has emerged from research that connects low birth weight to adult-onset illnesses. It suggests that malnutrition during early life could cause lasting changes that lead to negative effects much later in life. Exposure to harmful chemicals during fetal development may similarly have detrimental effects at maturity, as observed in the case of transplacental carcinogens^2^. Due to the time gap between prenatal exposure and the onset of adult cancer, the underlying causal effects must remain dormant for extended periods while remaining highly responsive to future stimulation. The characteristic traits of stem cells, such as quiescence, self-renewal, and immortality, enable the persistence of latent neoplastic cell populations throughout a person’s lifetime^3^. Fetal stem cells may indeed have a significant impact on transplacental carcinogenesis. The abundance of these cells has the potential to influence significantly the fetus’s sensitivity to carcinogens.

Inorganic As is a well-known transplacental carcinogen in rodents and is likely to have related effects in humans. Mice studies employing prenatal exposure suggest that As causes tumors or preneoplastic alterations in many organs, a pattern also seen in humans^4^. The idea that fetal stem cells are central targets in transplacental carcinogenesis is consistent with As’s potential to function as a multi-site carcinogen. While skin malignancies are common in individuals exposed to As, the mechanistic understanding of this process, particularly following prenatal exposure, is poorly understood. Investigations using in vitro models of human skin cancer have shown that As interferes with epidermal stem cells’ entry into differentiation pathways, disrupting their population dynamics. As increases the relative number of epidermal stem cells in vitro, indicating that they may be the primary target for in vivo carcinogenesis^5^. As cancer involves the deregulation of stem/progenitor cells, an As-induced increase in the quantity of skin stem cells or similar dysregulation in vivo could remain dormant while facilitating malignancy following further stimulation. Some studies indicate that As might transform healthy stem or progenitor cells into cancer stem cell (CSC)-like cells^6^. Moreover, interleukin-6 (IL-6) overproduction in the niche has been reported to boost the number of CSCs by directing surrounding stem cells towards a cancer phenotype in As-induced malignant epithelial cells^7^. However, many of these epithelial-centric studies have overlooked the potential role of microenvironment-forming stromal cell populations in the malignant conversion of stem cells.

Stem cells are susceptible and highly responsive to alterations in their microenvironment^8^, which provides necessary cues to regulate cell fate and tissue homeostasis. Deregulated crosstalk between fibroblasts in the stroma and stem cells can contribute to various pathological conditions, including fibrosis, impaired wound healing, and tumorigenesis. Under a pro-carcinogenic environment, stem cells may give rise to several tumor-aiding cells due to a disrupted self-renewal process, including the formation of CSCs^9^. CSCs maintain a significant part of tumor mass that orchestrate the fate of tumor progression under the influence of soluble factors and signaling molecules released by activated fibroblasts (AFs).

AFs are part of the stromal fraction with growth-promoting effects on SC/CSCs. However, it has yet to be discovered how precisely these cells affect the development and spread of epithelial malignancies ^10^. AFs arise from quiescent fibroblasts in response to multiple stimuli, while their clearance and restoration to a quiescent state in wound healing, senescence, and death are essential. However, chronic wound healing responses triggered by exposure to environmental carcinogens may cause irreversible fibroblast activation, likely associated with aggravated tumor progression ^11^. Fetal exposures to transplacental carcinogens like As may induce this activation by disrupting standard epigenetic settings. Fibroblasts may undergo a phenotypic transition in response to various stimuli, including prenatal carcinogen exposure. During this transition, fibroblasts upregulate markers such as α-SMA and secrete a range of pro-inflammatory cytokines, growth factors and extracellular matrix components to facilitate tumor development and invasion^12^.

Inflammation-promoting cytokines such as IL-6 in the environment surrounding tumors can activate skin cells and cause abnormal expansion. IL-6 is synthesized by various cell types, including AFs, CAFs, and cancer cells in the TME, promoting cancer spread and resistance to chemotherapy^13^. High IL-6 levels have been related to several skin conditions like psoriasis, hyperkeratosis, and scleroderma^13^.

Studies have revealed associations between specific epigenetic and circulatory inflammation markers. Alterations in epigenetic signatures may contribute to disturbed inflammatory response and an amplified risk towards chronic inflammatory diseases^14^. Epigenetic modifiers are essential in establishing and maintaining methyl marks that contribute to distinct cellular identities. The TET family is involved in several DNA modifications, including the conversion of 5-mC to 5-hmC, which can alter the normal chromatin state. Studies show that As exposure can contribute to changes in 5-hmC levels of several genes involved in developing and differentiating mouse embryonic stem cells^15^. Additionally, TET1 has been found to facilitate the demethylation of the promoters, including IL-6 gene, resulting in their increased production^16^. Knockdown of TET1 has been associated with reduced global DNA demethylation, indicating its essential role in DNA hypomethylation at gene promoters. In some cases of melanoma, the conversion of 5-mC to 5-hmC has been linked to immune pathway stimulation and infiltration of tumor-immune cells^17^. Although the participation of TET1 during innate immune responses has been described, the precise mechanisms by which TET1 regulates IL-6 expression in response to prenatal exposure remain unclear.

We have previously demonstrated that exposure to As during prenatal development aggravates tumor response in a two-stage cutaneous carcinogenesis model by increasing proliferating epidermal stem cells^18^. In this study, we have explored how Keratinocyte stem cells (KSCs) are modulated following prenatal As exposure and the contribution of As -conditioned fibroblasts in establishing a pro-tumorigenic niche. Following prenatal As exposure, we isolated fibroblast cells from offspring and cultivated unexposed KSCs in the fibroblast-conditioned media to assess the effects and identify the factors responsible. Our study identifies the factors responsible for increased fibroblast activation and their pro-tumorigenic effects on KSCs.

## Materials and procedures

Sodium (meta) arsenite was procured from Sigma Aldrich, USA. Bio-Plex Pro™ mouse cytokine 5-plex magnetic bead panel from Biorad, USA. DeadEndTM Colorimetric TUNEL System kit was obtained from Promega, USA. MeDIP grade antibodies (5-hmC, 5-mC) were obtained from Abcam in the UK. Protein A/G beads were purchased from Santa Cruz Biotechnology, Inc., in the USA. For qPCR analysis, the primers were designed by Eukaryotic Promoter Database (EPD) and obtained them from IDT Technologies, India. Supplementary Table 1 contains a list of the chemicals and antibodies used with their respective sources.

### Animals and experimental treatments

All animal experiments adhere to the ARRIVE guidelines, and the experimental protocols were executed with approval from the Institutional Animal Ethical Committee under the reference number IITR/IAEC/29/17-38/2018. BALB/c female mice, aged five weeks, were acquired from the animal rearing facility at the CSIR- Indian Institute of Toxicology Research. The mice were randomly separated into two groups after one week of acclimatization. The animals received unlimited access to food and fresh water. Standard laboratory settings for animal care included 12-h light/dark cycles and 25 °C room temperature. A freshly prepared dose of sodium (meta) arsenite with a fixed concentration of 0.04 mg/kg As was given orally from 15 days prior to mating (GD-15) until the pups were born. Oral dose administration was chosen as it has previously been established that *dams* may lower their water consumption due to exposure stress^19^, which might further alter the litter size. Exposure duration to female mice was followed, considering OECD guidelines for developmental toxicity studies. Two-stage cutaneous carcinogenesis model was followed from 6 to 18 weeks. Animals were euthanized using a ketamine overdose at all experimental endpoints. Also, PND 2 pups were obtained from all groups after delivery. In vitro experiments were carried out after the skin cells were cultured from the pups. Primary DFs were extracted from control and prenatally exposed pups; primary KSCs were isolated from only unexposed control pups.

### Primary DFs culture

Following delivery, pups were sacrificed to isolate skin tissues at PND 2. The tissues were allowed to float on trypsin (w/o EDTA) with the epidermal layer facing down for 1.5 h placed at 37 °C to digest the epidermis. The dermal layer was thinly sliced using a sharp scalpel and was filtered by a 70 µm filter twice in cold DMEM-CM (DMEM high glucose—Complete Media). The cells were centrifuged at 180 xg for 10 min maintaining 4 °C to form a pellet. The cells were next seeded and cultivated in DMEM-CM till 70% confluence and passaged 3 times (P3) afterwards. All the experiments were conducted at P3.

### Media conditioning

After 70% confluence at P3, DFs were checked for purity via positive Vimentin and Pdgfrα staining, more than 95% positively stained cells confirmed cell purity. DFs were treated with mitomycin for 1.5 h to arrest their growth. Growth arrest was further ensured by replenishing DMEM-CM overnight. When no changes in growth were observed, the cells were used to condition EMEM-GM (EMEM-Growth Media) overnight (16–18 h). The DF-conditioned EMEM (EMEM-CM) was mixed in a ratio of 1:1 with unconditioned EMEM-GM. This media was further used to culture unexposed KSCs.

### Primary KSCs culture

At PND 2, unexposed pups were sacrificed. Whole skin was removed after the excision of the limbs and tail. Excessive fat was removed from the dermal side. Whole skin tissues were placed to float over cold trypsin (w/o EDTA) solution with the dermal side down following an incubation of 16 h at 4 °C to attain efficient digestion of the dermis. The subsequent day, the epithelial sheet was separated from the digested dermal layer to isolate KSCs. The separated epithelial sheet was finely minced and passed via a 40 µm filter to generate a single-cell suspension. After that, the suspension was centrifuged at 180 × g for 8 min. Resulting pellet was resuspended in a 1:1 mixture of EMEM-GM and EMEM-CM (DF-conditioned EMEM). The cells were further cultivated at 37 °C with 5% CO_2_, and fresh media (in 1:1 ratio) was provided every other day. The purity of the cells was assessed by performing dual staining for CD34 and Integrin α6.

### Immunofluorescence and immunocytochemistry

Tissue immunofluorescence (IF) analysis was conducted on skin sections acquired at PND 2 and 18 weeks to examine the expression levels of cell-specific markers. Immunocytochemistry (ICC) was used to assess activation in primary DFs. The cells were fixed for 10 min in ice-cold paraformaldehyde (4%) and then stained with antibodies against α-SMA, Collagen IV and Fibronectin. Similarly, STAT3 cascade activation in KSCs was evaluated using antibodies against GP130, pJAK2, and pSTAT3. Goat anti-rabbit IgG conjugated with Cy3 was used to detect the primary antibodies. 4′,6-diamidine-2′-phenylindole dihydrochloride (DAPI) was used to stain cell nuclei and DNA. Fluorescence images were acquired by NIKON inverted confocal microscope. Analysis for nuclear markers is done by counting positively stained nuclei per high power field (HPF). Staining intensities of cytosolic markers are reported as mean fluorescence intensity (MFI), measured by ImageJ software.

### Clonogenic assay

Unexposed primary KSCs were distributed across multiple 6-well plates having the density of 500 cells per well. Following an overnight incubation period the cells were cultivated and maintained in a controlled environment at 37 °C with 5% CO_2_ for duration of 14 days. The fibroblast conditioned medium was refreshed every 2 days during this period. Afterward, the cells were fixed and their colony appearances were assessed. To fix the colonies, 70% ethanol was used, and they were subsequently stained with 0.5% crystal violet solution. A cell colony was identified as a group of at least 50 cells and quantified using Image J software. Images are acquired by ChemiDoc™ MP (BioRad), version 2.4.0.03.

### Scratch assay

The migratory capacity of KSCs cultivated in prenatally exposed dermal fibroblasts conditioned media (EMEM-CM) was examined in vitro using the scratch assay. A wound was created by scratching the cell monolayer with a p200 microtip to produce the effects of an injury. The migration was tracked for a period of 18 h. The healed area was measured using ImageJ software. The graph represents the area healed vs. time.

### BrdU labeling

Unexposed primary KSCs were cultivated in EMEM-CM and cultured at 37 °C in a humidified 5% CO_2_ atmosphere. After plating, the cells were pulse-labeled with a 10 µM BrdU labeling solution in the medium for 12 h. The DNA was hydrolyzed by 1 N HCL for 15 min at room temperature, following an incubation of 5 min in a neutralizing buffer (0.1 N sodium borate, pH 8.5). Next, the cells were rinsed with PBS and fixed for 10 min in cold paraformaldehyde (4%). Cells were then checked for BrdU incorporation using anti-BrdU antibody following standard immunocytochemistry protocols. At 100× magnification, the mean percentage of positive cell counts to the total cell population was calculated and used to assess in vitro KSC proliferation activity induced by conditioned media.

### Immunoblotting

Cells were lysed in RIPA buffer (100 mmol/l dithiothreitol, 50 mmol/l Tris–HCl, pH 6.8, 2% SDS, and 10% glycerol) with protease inhibitors. The amount of total protein present in the samples was quantified by BCA (bicinchoninic acid protein) assay. Following protein separation by SDS-PAGE, the gels were transferred to a PVDF membrane. The membranes were incubated in blocking buffer containing 0.05% Tween 20 and 5% non-fat milk in TBS for 1 h to avoid unspecific binding. The membranes were treated with primary antibodies for 16–18 h at 4 °C after blocking. The membranes were subjected to the appropriate HRP-conjugated secondary antibodies for 2 h after three TBST washes. The protein quantity was assessed by normalizing it with GAPDH, while pSTAT3 was normalized in relation to total STAT3. Protein bands were visualized using ChemiDoc™ MP (BioRad), version 2.4.0.03. Consistent exposure settings were applied to capture all images within a particular experimental set. Uncropped blots for respective figures have been provided in supplementary Fig. 1.

### RNA extraction and cDNA synthesis

KSCs grown in EMEM-CM were harvested for total RNA using Trizol. Following that, 1 ug of the total RNA was reverse-transcribed into cDNA using High capacity cDNA reverse transcription kit. Quantitative Real-Time PCR was performed in triplicate using SYBR qPCR Master Mix and a Quant 6 Flex Real-Time PCR System. The PCR settings included a first step lasting 10 min at 95 °C, following 40 amplification cycles, lasting 15 s at 95 °C and 60 °C, respectively. *Gapdh* was used as an internal reference for normalization in order to measure the levels of gene expression using the comparative Ct approach. Supplementary Table 2 lists the primer sequences used for PCR.

### Multiplex-based assessment of selected genes

Cytokine levels in primary DFs were assessed using a bead-based MAGPIX Multiplex Reader (Bio-Rad Laboratories, Hercules, CA). Cell lysate and enriched supernatant were obtained from both the control and prenatally exposed DFs after overnight incubation. To summarize, the magnetic assay plate underwent a series of washes with a shaker at 500 rpm, using a wash buffer. Subsequently, 25 μl of magnetic beads coated with specific antibodies for different cytokines were added to each well of a 96-well magnetic assay plate and mixed briefly. After removing the liquid, the plate was washed three times with wash buffer using a hand-held magnet to eliminate any unbound beads and other soluble factors. Next, 50 μl of the sample was added to the wells containing the magnetic bead-coated antibodies and left to incubate at room temperature for 1 h on a plate shaker at 500 rpm. Following this incubation, the liquid was removed, and the plate was washed (3×) with wash buffer using the hand-held magnet to remove any remaining unbound substances. Biotinylated detection antibodies (25 μl) were introduced into each well and incubated at room temperature for 45 min with shaking (500 rpm). After this incubation, 25 μl of streptavidin-PE was added to each well containing the biotinylated detection antibody and further incubated at room temperature for 10 min.

Finally, the plate was washed (3×) with wash buffer, and 120 μl of reading buffer was added to each well. To read the plate, we used Luminex MAGPIX multiplex reader from Bio-Rad. The levels of cytokines were determined by measuring the infrared (IR) fluorescence associated with various antibody-coated magnetic beads. The results were presented as the mean fluorescence intensity (MFI) relative to the control group. Six cytokines (IL-1β, IL-1α, IFN-γ, TNF-α, MCP-1, and IL-6) secreted by DFs, known to affect the proliferation and survival of KSCs were examined (Supplementary Fig. 2B). IL-6 was the sole cytokine notably present in the supernatant of DFs exposed prenatally to arsenic (Fig. 3C).

### MeDIP analysis

MeDIP experiments were conducted following previously outlined protocol ^20^. Total DNA was isolated using the standard PCI method. 1 µg of purified DNA suspended in TE buffer (pH: 8.0) (120 µl) was sheared (Bioruptor™ UCD-200, Diagenode) to obtain a fragment size of 200–1000 bp. Sonicated DNA fragments were denatured for 10 min at 95 °C. Next, the DNA was ice-cooled for 10 min immediately, followed by incubation with 5-mC and 5-hmC separately in cold NP-40 buffer with overhead shaking for 16–18 h at 4 °C. DNA-antibody complexes were incubated with protein A/G beads (Thermo Fisher) on a rotor at 4 °C for 4 h. Following washing by NP-40 buffer (3×), the complex was next digested in proteinase K buffer [proteinase K (10 mg/ml) in TE buffer] at 56 °C for 2 h. DNA was next purified for further analysis by qPCR. Primers were designed following the previous protocol ^20^. The methylation status at the promoter region is represented by drawing the ratio of signals in immunoprecipitated DNA concerning input samples (sonicated single-stranded DNA). See Supplementary Table 2 for primer sequences.

### Statistical evaluation

GraphPad Prism version 8.0 was used to do statistical evaluation on the raw data. When comparing two experimental groups, data analysis was done using the unpaired, two-tailed Student’s t-test. Two-way ANOVA and Tukey tests were implemented on datasets with four experimental groups. The figure legends provide a list of all statistical tests that were conducted together with the accompanying n (sample size) and p values.

### Ethics approval

All the animal experiments were conducted after prior approval of Institutional Animal Ethical Committee (Approval no. IITR/IAEC/29/17-38/2018). The institutional animal ethics Committee follows the guidelines of the Committee for Control and Supervision of Experiments on Animals (CCSEA), Ministry of Fisheries, Animal Husbandry and Dairying (MoFAH&D), Govt. of India which regulates all animal experiments in India.

## Results

### Prenatal arsenic exposure disrupts EMT-associated proteins in the skins of offspring

**Figure 1:**
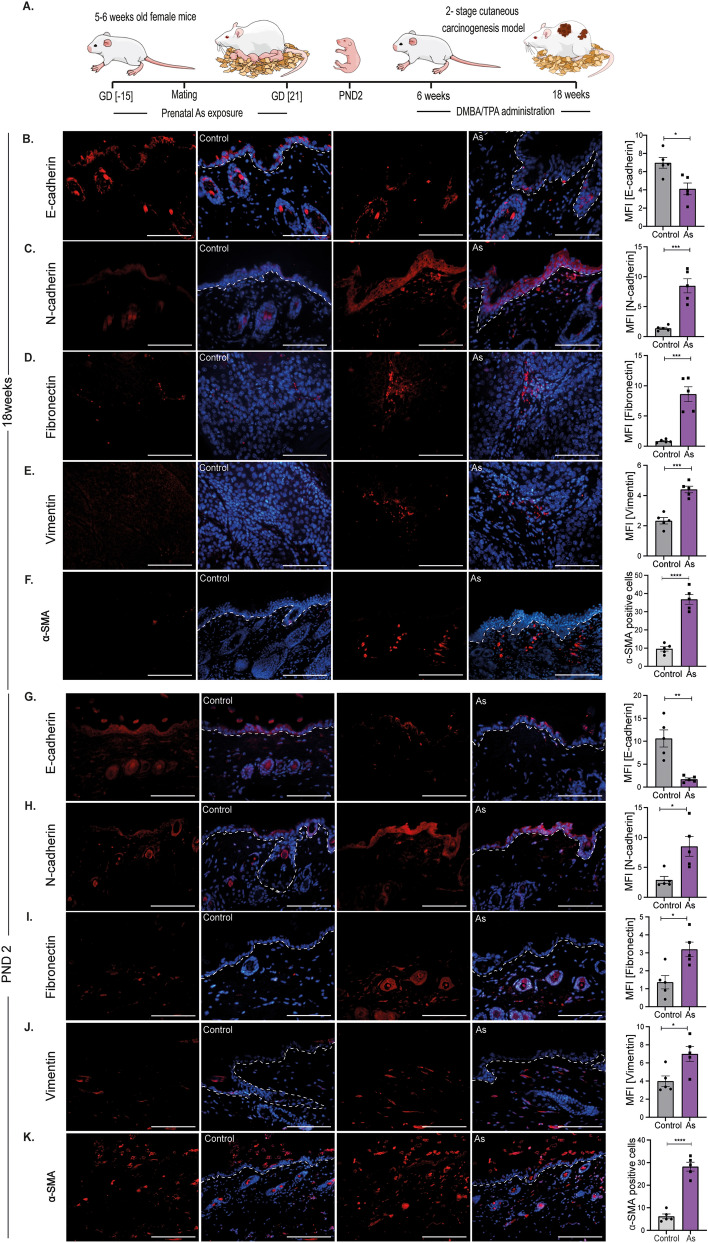
Prenatal arsenic exposure promotes EMT in mouse skin. (**A**) The figure depicts the experimental schedule, where mice were exposed to arsenic from 15 days before mating and throughout gestation until GD 21. Subsequently, the offspring received DMBA/TPA treatment, commencing at 6 weeks of age and persisting until 18 weeks. Assessment of EMT was done in skin tissue sections of offspring at 18 weeks (**B**–**F**) and PND 2 (**G-K**). Representative images showing the level of E-cadherin, N-cadherin, Fibronectin, Vimentin, and α-SMA proteins involved in EMT; n = 5; Scale = 50 µm. The respective graph shows positive staining intensities presented as MFI; α-SMA expression is presented as positive cell counts per field; n = 5; Scale = 100 µm. Error bars indicate mean ± SEM. The significance levels were determined as *p < 0.05, **p < 0.01, and ***p < 0.001 using an unpaired Student's t-test (two-tailed).

Our previous study suggested that only prenatal As exposure sufficiently enhances the effects of 7, 12-Dimethylbenz[a]anthracene/12-*O*-Tetradecanoylphorbol-13-acetate (DMBA/TPA), resulting in increased tumor numbers with reduced latency^18^. Tumors developed under the effects of prenatal exposure contained basal cell carcinoma (BCC) and squamous cell carcinoma (SCC) types, with increased levels of PCNA, MMP 9, and Vimentin at 18 weeks. In this study, we hypothesized that prenatal As exposure might induce EMT in the skins of the offspring at an early stage which may lead to the development of aggressive tumors when re-challenged with DMBA/TPA. Following the same experimental regimen, we validated if EMT markers are elevated in response to prenatal exposure. EMT proteins, including E-cadherin, N-cadherin, Vimentin, and Fibronectin, were checked at 18 weeks (Fig. 1B–E) and postnatal day 2 (PND 2) (Fig. 1G–J). At termination, i.e., 18 weeks EMT proteins including E-cadherin, N-cadherin, Vimentin and Fibronectin were checked in tumour sections. In prenatally exposed animals, a decrease in the levels of E-cadherin (MFI 4.1 ± 0.6), with increase in N-cadherin (MFI 8.5 ± 1.2), Vimentin (MFI 4.4 ± 0.2) and Fibronectin (MFI 8.6 ± 1.2) was observed in comparison to control group animals with the expression levels in E-cadherin (MFI 7.0 ± 0.5), N-cadherin (MFI 1.4 ± 0.2), Vimentin (MFI 2.3 ± 0.2) and Fibronectin (MFI 0.8 ± 0.1). Further, to validate if these markers were elevated in response to prenatal exposure, before receiving any secondary hit, we checked these markers at an early stage, i.e., PND 2. The results confirmed an early EMT switch due to prenatal exposure as observed by the low levels of E-cadherin (MFI 1.7 ± 0.3) compared to control mice (MFI 11 ± 1.9). Also, upregulation in the expression of N-cadherin (MFI 8.5 ± 1.6), Vimentin (MFI 7.0 ± 08) and Fibronectin (MFI 3.2 ± 0.4) was clear with reference to control animals, i.e., (MFI 2.9 ± 0.5), (MFI 4.0 ± 0.5) and (MFI 1.4 ± 0.3) respectively.

### Prenatal arsenic exposure induces dermal fibroblast activation in mice

Our previous findings depicted disturbed basal cell dynamics, including increased proliferative rate, further causing accelerated tumorigenesis in the offspring. As basal cells are actively dividing progenitors, there may be some discrepancies in the functions of parent KSCs. In skin malignancies, stem cells may acquire EMT properties under the modified niche environment. As DFs play an essential role in stem cell niche modification during wounding and tumor conditions, we checked for their activation, comparing α-SMA positive cells in the dermis of prenatally exposed and control mice both at 18 weeks (Fig. 1F) and PND 2 (Fig. 1K). At 18 weeks, prenatally exposed offspring showed higher positive cells (26.2 ± 2.6) than control offspring (6.2 ± 1.0). Similarly, at PND 2 As group demonstrated a rise in α-SMA positive cell counts (37 ± 2.7) compared to unexposed pups (9.6 ± 1.2). These observations inferred a rise in AFs in prenatally exposed offspring at PND 2 and 18 weeks.

**Figure 2:**
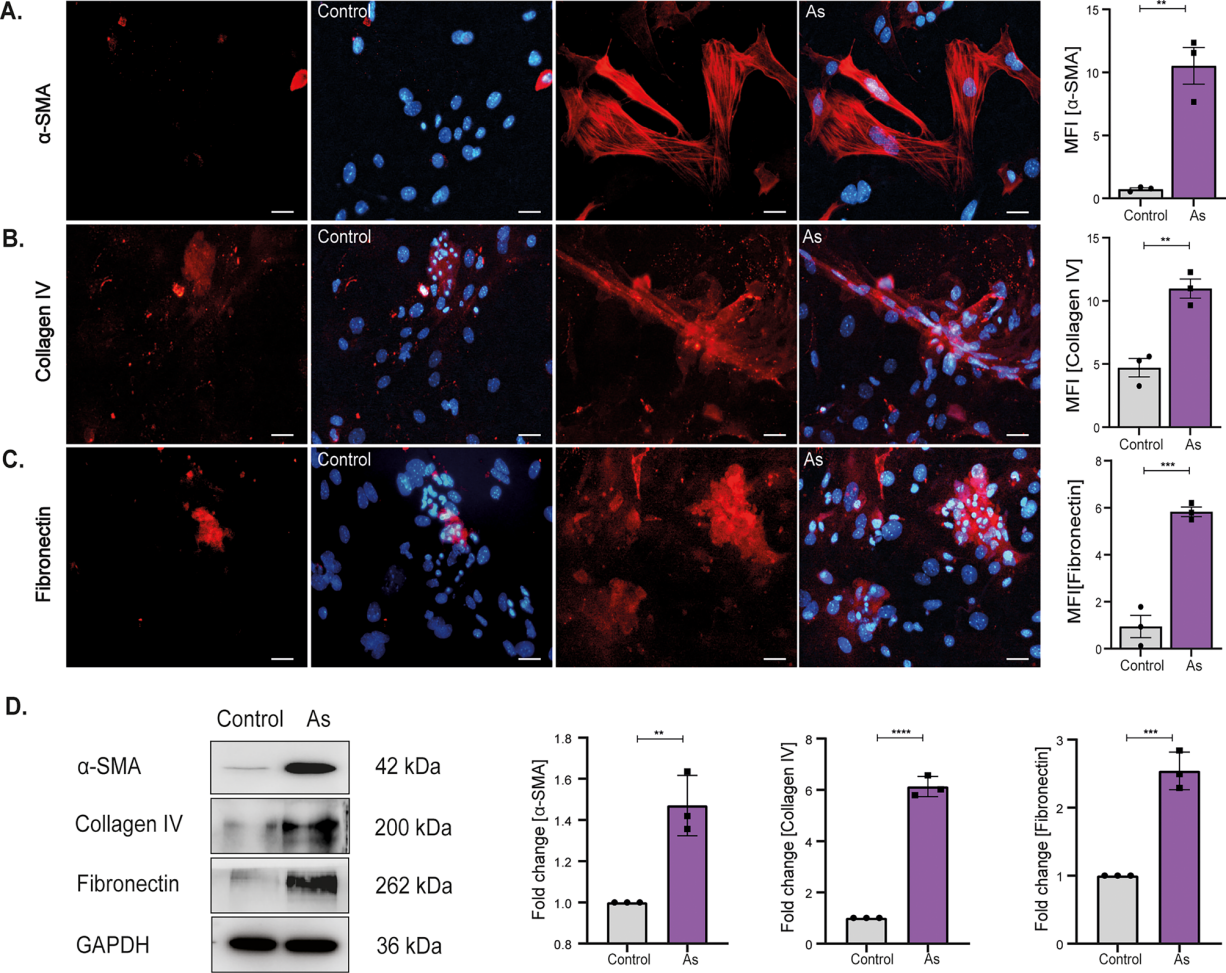
Prenatal arsenic exposure promotes the transition of DFs into activated phenotypes in vitro. The transition of DFs induced by prenatal exposure was assessed on primary cells isolated at PND 2 (**A**–**C**). Representative images for EMT proteins, α-SMA, Collagen IV, and Fibronectin are presented. An increased number of α-SMA-stained stress fibers signify the differentiation of DFs into AFs. Graphical representation of MFI in prenatally exposed cells compared to control cells. (**D**) Immunoblots represent the quantitative analysis of total EMT proteins in cultured cell lysates. Error bars represent mean ± SEM; n = 3; Scale = 50 µm. Significance calculated as *p < 0.05; **p < 0.01; ***p < 0.001; ****p < 0.0001 by unpaired Student’s T-test (two-tailed).

Activation of fibroblasts was also confirmed in vitro (Fig. 2). Following prenatal As exposure, DFs were isolated at PND 2 and were checked for their transformation into AFs by cell-specific markers, α-SMA, Collagen IV, and Fibronectin. Cultured fibroblasts showed a high number of α-SMA (MFI 10.5 ± 1.4) positive cells (Fig. 2A) in prenatally exposed group compared to the control (MFI 0.7 ± 0.1) cells. Additionally, the formation of stress fibers is responsible for mediating contractile function, demonstrating induced activation of fibroblasts in prenatally conditioned cells. AFs are characterized by increased synthesis of ECM (Extra Cellular Matrix) components, including stromal collagens.

A significant increase in the levels of Collagen IV (MFI 10.9 ± 0.7) concerning control cells (MFI 4.7 ± 0.7) confirmed induced activation of fibroblasts caused by prenatal stress (Fig. 2B). Another ECM protein, Fibronectin, known to be strongly involved in metastasis, was observed to be upregulated (MFI 5.8 ± 0.8) in prenatally conditioned fibroblasts compared to control cells (MFI 0.9 ± 0.4) (Fig. 2C). These results were also validated in whole cell lysates by immunoblotting (Fig. 2D), where levels of total proteins α-SMA, Collagen IV, and Fibronectin increased by 1.4 ± 0.08, 2.4 ± 0.2, 2.2 ± 0.1fold respectively.

### Prenatally exposed DFs promote colony formation and EMT in KSCs via IL-6-enriched secretome

**Figure 3:**
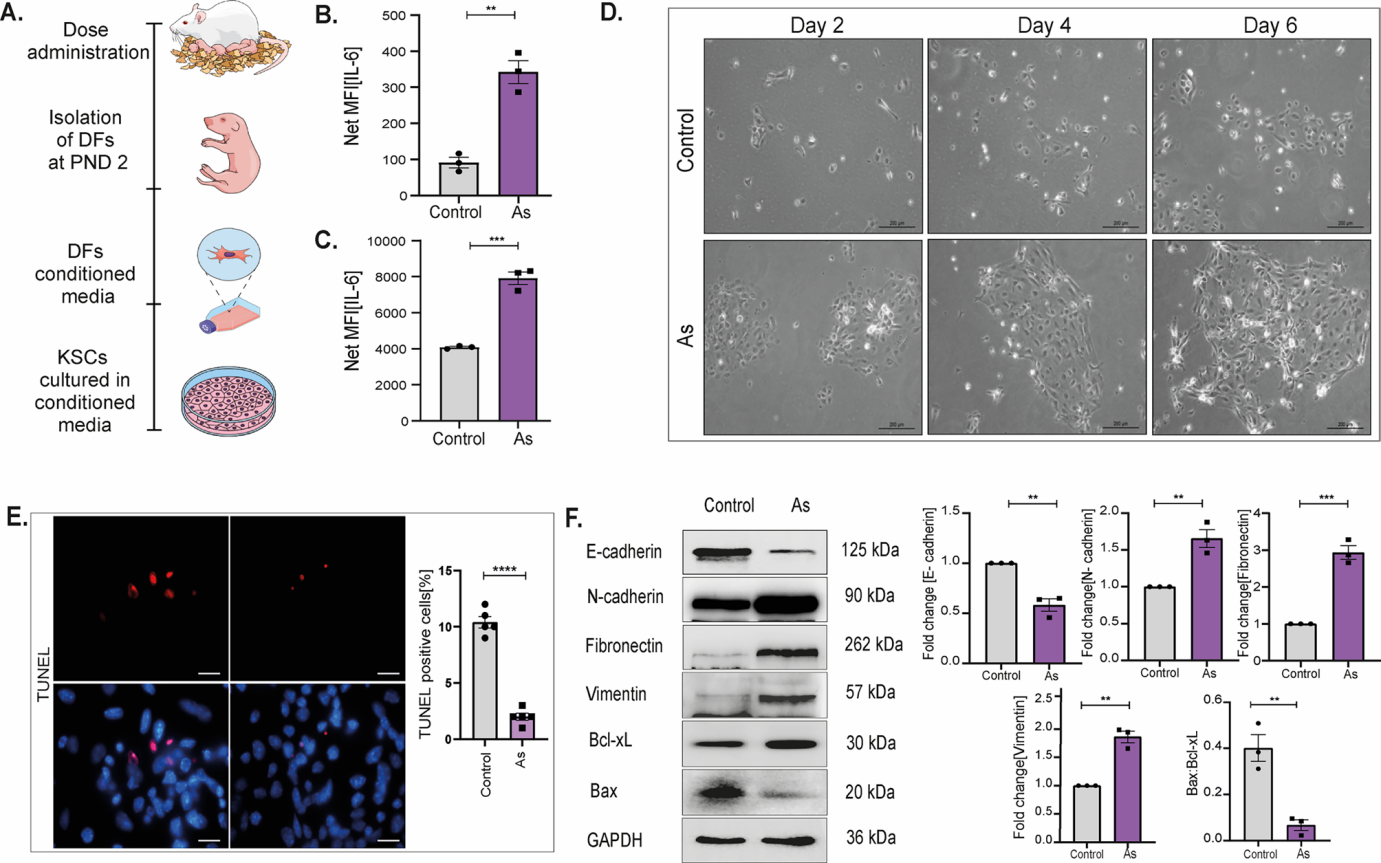
Activated DFs derived IL-6 rich secretome induces survival and EMT in KSCs. Both prenatally exposed DFs and unexposed KSCs were isolated from the offspring at PND 2 for in vitro experiments. (**A**) Primary DFs were isolated from the F1 generation at PND 2, followed by conditioning the media and culturing unexposed primary KSCs in it. The expression of IL-6 in both cell (**B**) lysate and (**C**) supernatant of prenatally exposed DFs was measured by ELISA assay and is reported as net MFI; n = 3. (**D**) Representative images display colony formation in KSCs cultured in DF-CM on days 2, 4, and 6, captured by phase contrast microscope; scale = 200 µm. (**E**) Survival in KSCs was quantified by determining the percentage of TUNEL positive cells per field. (**F)** Immunoblot quantification of EMT markers in cultured KSCs displays protein levels of N-cadherin, Vimentin, Fibronectin, and E-cadherin in IL-6 enriched EMEM-CM. The Bax to Bcl-xL ratio depicts the survival rate in KSCs. Graphs represent the quantification of total proteins in cell lysates of primary KSCs; n = 3. Error bars in all graphs denote the mean value with standard error of the mean (SEM). Statistical significance was calculated using an unpaired Student’s T-test (two-tailed) and is indicated as *p < 0.05, **p < 0.01, ***p < 0.001, ****p < 0.0001.

Cancer-associated stroma produces and releases diverse proteins, including IL-6, that promote tumor development and invasion^21^. Figure 3A represents islolation of primary DFs at PND 2. These fibroblasts are then subjected to a process of media conditioning, and subsequently, keratinocyte stem cells are cultured in this conditioned media. To assess the degree of expression and cellular source of IL-6, we used ELISA to identify its expression in both lysate and supernatant acquired from cultured DFs. As indicated in Fig. 3B, IL-6 levels in the prenatally exposed cell lysates were considerably higher (MFI 342.2 ± 31.6) than in unexposed control cells (MFI 91 ± 14.4). Similarly, IL-6 expression was substantially greater in cell supernatant procured from previously conditioned cells (MFI 7919 ± 350.4) vs. control cells (MFI 4086 ± 50.4) as presented in Fig. 3C.

IL-6 is a known promoter for EMT and proliferation in epidermal stem cells. Since prenatal As exposure induced a high synthesis and release of IL-6 in DFs, we used this secretome to study niche modifications in unexposed primary KSCs. The first morphological difference observed was the time difference in colony formation between KSCs cultured with and without prenatally exposed dermal fibroblasts conditioned media. Media conditioned over prenatally exposed DFs induced a faster colony formation in KSCs than those cultured in media from unexposed fibroblasts. The snapshots were taken focusing same fields; representative images of days 2, 4, and 6 post-cell seeding are shown in Fig. 3D. In tumorigenesis, stem cell survival is an important aspect; therefore, the survival rate of KSCs was examined by TUNEL assay (Fig. 3E). Representative images demonstrate a reduced number of TUNEL-positive (apoptotic) cells in KSCs with prenatally exposed conditioned media (2 ± 0.3%) vs unexposed cells (10.4 ± 0.5%), showing a high survival rate acquired by KSCs induced by activated DFs.

Unexposed KSCs were cultured in conditioned media to examine the role of prenatally AFs in mediating EMT in skin tumors. As shown in Fig. 3F, the culture of KSCs in EMEM-CM acquired from prenatally exposed cells significantly reduced E-cadherin expression by 0.5 ± 0.06 fold while increasing N-cadherin expression by 1.6 ± 1.2 fold, confirming an alteration in their microenvironment. Also, cell marker Vimentin and Fibronectin, responsible for migration, were upregulated by 2.9 ± 1.8 and 1.8 ± 0.1 fold, respectively. The survival rate in KSCs cultured in EMEM-CM was also checked by drawing Bax to Bcl-xL ratio. A reduced ratio validated a high survival rate in these cells.

### Activated fibroblasts derived IL-6 enriched conditioned media promotes GP130/JAK2/STAT3 activation in KSCs

The conventional IL-6 signal transduction pathway begins with interaction with IL-6R and phosphorylation of STAT3 through JAK2. To investigate the involvement of the IL-6/JAK2/STAT3 pathway in AFs-induced EMT alterations in KSCs, we first studied the activation of the IL-6/JAK2/STAT3 pathway in KSCs following their culture in conditioned media.

**Figure 4:**
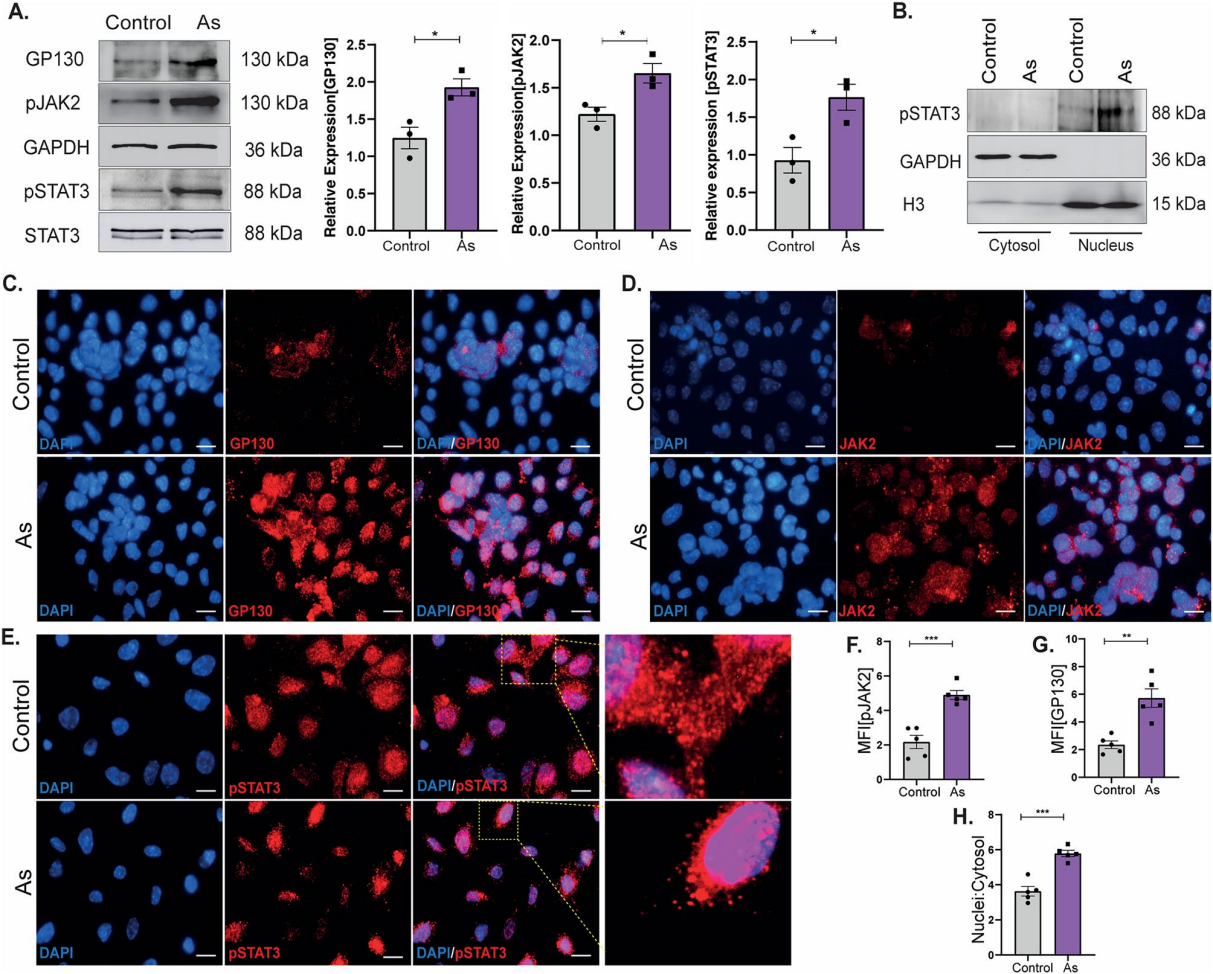
Prenatally activated DFs stimulates JAK2-STAT3 signaling in KSCs. Evaluation of IL-6/STAT3 pathway was done by western blotting and ICC. (**A**) Total proteins were measured by western blot analysis to confirm activated pathway. Graphical representation of densitometry quantification show IL-6/STAT3 pathway proteins; n = 3. (**B**) Immunoblots showing pSTAT3 expression in nuclear extracts of KSCs. GAPDH and Histone 3(H3) represent cytosolic and nuclear controls respectively. (**C**–**E**) ICC images of GP130, pJAK2, and pSTAT3 in KSCs cultured in EMEM-CM. Images indicate an increased nuclear translocation of pSTAT3 in KSCs cultured with prenatally conditioned DF-CM. (**F**, **G**) Graphs represent the Mean Fluorescence Intensity (MFI) of cell-specific markers and nuclei to cytosol ratio (**H**) for pSTAT3 translocation, with a sample size of n = 5. The scale bar in ICC images corresponds to 50 µm.

An essential component of the IL-6 receptor is GP130, which is needed for the receptor’s proper functioning, allowing its downstream cascade to be activated. Activation of the JAK-STAT pathway was validated at protein levels with upregulated levels of GP130 (1.7 ± 0.1), JAK2 (1.6 ± 0.1), and STAT3 (1.6 ± 0.1) in KSCs cultured in prenatally exposed conditioned media (Fig. 4A). ICC results in Fig. 4C–H also demonstrate upregulated levels of GP130, confirming an overexpression of IL-6 receptors in KSCs. Also, high phosphorylated protein levels of JAK2 and STAT3 established the activation of this pathway in KSCs cultured in prenatally exposed EMEM-CM.

Under basal conditions, STAT3 may be found in both the cytoplasm and nucleus, which shows that STAT3 constantly moves between the two cellular compartments. Activation of STAT3 involves multiple steps, including phosphorylation by JAK2, followed by the dimerization of pSTAT3 and translocation in the nucleus, DNA binding, and expression. Increased import of STAT3 in the nucleus has been implicated in cancers as it activates genes specific to cell cycle progression, EMT, and survival. Increased translocation was confirmed by protein levels of pSTAT3 in cytosolic and nuclear fractions of KSCs (Fig. 4B). These results were also validated by ICC results (Fig. 4E, H). pSTAT3 was observed to be localized more in the nuclei of KSCs than in cytoplasmic regions, providing evidence of active translocation due to dysregulated niche.

### Blockade of GP130/JAK2/STAT3 pathway impairs proliferation, migration, and EMT in KSCs

We investigated the role of the IL-6-induced GP130/JAK2/STAT3 pathway in the proliferation, migration, and EMT of KSCs, as these properties are known to promote cell metastasis in cancer. We confirmed the influence of AFs in modifying the colony-forming potential, proliferation, migration, and EMT properties of unexposed KSCs.

**Figure 5:**
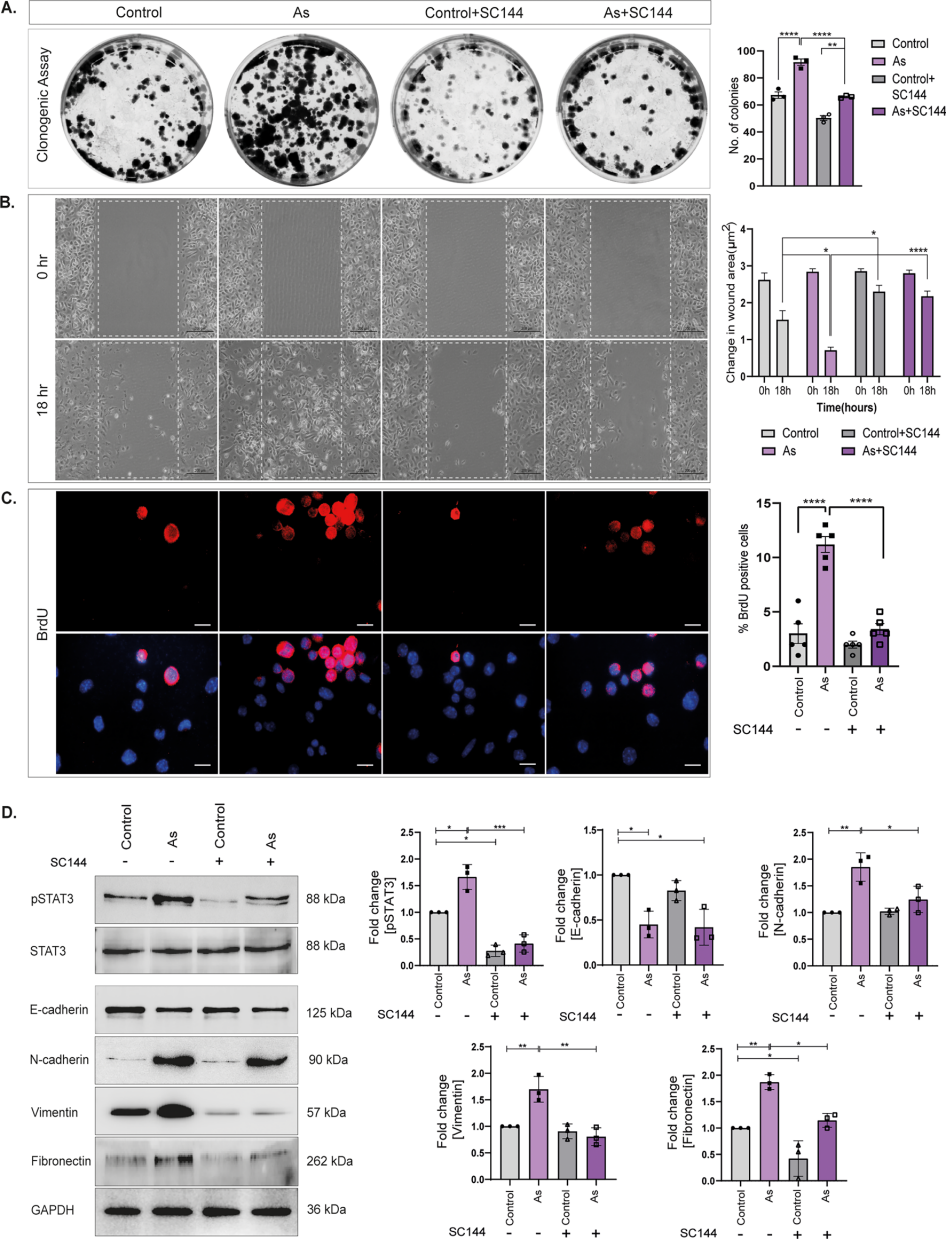
Blocking IL-6-STAT3 activation impairs colony formation, proliferation, and migration in KSCs. Acquired proliferative and migratory properties in KSCs were evaluated by clonogenic, scratch wound and BrdU assay. (**A**) Representative images of clonogenic assay showing before and after pSTAT3 blockade in KSCs. Images were captured using ChemiDoc™ MP (BioRad). (**B**) Representative images of scratch wound assay at 18 h in KSCs with EMEM-CM, before and after pSTAT3 inhibition. (**C**) BrdU proliferation assay showing a number of dividing cells in KSCs cultured in EMEM-CM before and after pSTAT3 inhibition by SC144. Graphs represent the quantification of respective assays; n = 5; scale = 200 µm. Error bars represent mean ± SEM. *p < 0.05, ****p < 0.0001 according to two-way analysis of variance (ANOVA) and Tukey’s multiple comparison tests. (**D**) Progression of EMT in KSCs validated by immunoblotting. Total protein separated and developed against pSTAT3, N-cadherin, Vimentin, Fibronectin, and E-cadherin. Graphs show quantification of total protein levels in primary KSCs; n = 3. **p < 0.01; ***p < 0.001; ****p < 0.0001 computed using two-way ANOVA and Tukey’s multiple comparison tests.

To validate these findings, we used SC144, a specific inhibitor of IL-6-induced STAT3 activation, to block the activation of phosphorylated STAT3 (pSTAT3). Colony forming ability in KSCs was checked by clonogenic assay (Fig. 5A) where number of colonies in conditioned group (As) were ≥ 92 compared to control with colony no. ≥ 67. However, the colony formation rate declined after pSTAT3 inhibition in both control and As groups, i.e., ≥ 50 and ≥ 66, respectively. Figure 5B illustrates high migratory rate of KSCs, as seen in representative images taken at 18 h, where complete healing of the wound was observed in KSCs cultured in prenatally exposed DFs culture media. Increased migration was further reduced after inhibiting pSTAT3, confirming the involvement of activated STAT3 signaling.

The proliferative rate induced in KSCs by EMEM-CM was validated by BrdU assay in primary KSCs (Fig. 5C). The results depicted an increased uptake of BrdU by KSCs demonstrating a high division rate (11.2 ± 0.7%) in these cells when cultured in IL-6-rich media vs. control cells (3 ± 0.8%). However, the number of BrdU positive cells reduced significantly after pSTAT3 inhibition confirming induction of proliferation of KSCs (3.4 ± 0.5%) than in control (2.0 ± 0.3%) KSCs due to activated IL-6- STAT3 axis following prenatal As exposure.

To confirm the involvement of the IL-6-STAT3 axis in promoting EMT, we re-evaluated the protein levels after inhibiting pSTAT3 with SC144 (Fig. 5D). We observed an upregulation of pSTAT3 by 1.7 ± 0.1 fold in KSCs cultured in prenatally conditioned media. However, the expression decreased to 0.41 ± 0.09 after inhibition with SC144, compared to 0.28 ± 0.06 in the control group treated with SC144. The levels of EMT proteins were also rescued after pSTAT3 inhibition, confirming the role of IL-6-induced activation of the JAK2-STAT3 pathway in KSCs. The observed levels of E-cadherin in the prenatally exposed group were 0.4 ± 0.08 fold, with no significant changes after pSTAT3 inhibition, i.e., 0.43 ± 0.03 fold. Similarly, the upregulated levels of N-cadherin, which were 1.8 ± 0.1 fold in KSCs cultured in prenatally conditioned fibroblast media, decreased to 1.2 ± 0.14 fold. The protein levels of Vimentin also decreased from 1.7 ± 0.14 fold in the prenatally exposed group to 0.8 ± 0.09 fold in the group treated with both prenatally conditioned media and SC144. Fibronectin levels decreased from 1.8 ± 0.08 to 1.1 ± 0.07 fold after inhibiting the IL-6-STAT3 cascade.

### TET1 increases IL-6 production by the accumulation of 5-hmC at the promoter in prenatally exposed primary DFs

**Figure 6:**
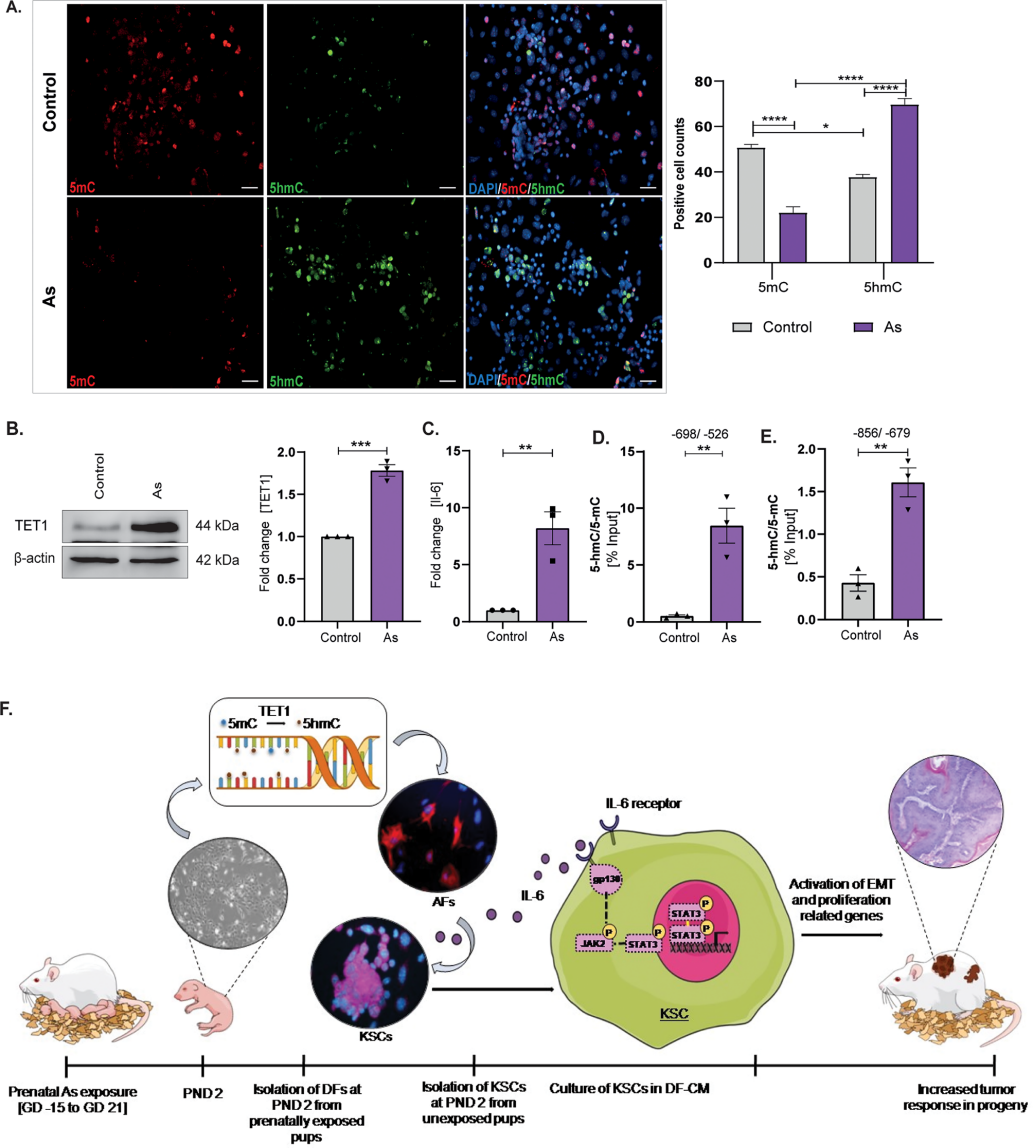
TET1 mediates IL-6 overexpression via increased 5-hmC accumulation at the gene promoter. MeDIP analysis of 5-hmC and 5-mC at IL-6 promoter for TET1 activity. (**A**) Representative images show conversion of 5-mC to 5-hmC in prenatally exposed DFs. The graph represents numbers of 5-hmC and 5-mC positive cells in primary DFs. (**B**) Immunoblot analysis of total TET1 protein in primary DFs. (**C**) mRNA levels of IL-6 in prenatally exposed primary DFs presented as fold change. (**D**, **E**) MeDIP results are represented as a 5-hmC: 5-mC ratio adjusted to input. Results are reported as relative enrichment (n = 3) with respect to the control group covering two promoter sites. Scale bars = 50 µm. Data are presented as the mean ± SEM. Significance calculated as *p < 0.05; **p < 0.01; ***p < 0.001 by an unpaired Student’s T-test (two-tailed). (**F**) Schematic summary depicting the impact of prenatal As exposure on increased skin carcinogenesis. Prenatal exposure results in an increased expression of TET1 in dermal fibroblasts. This leads to a simultaneous rise in 5-hmC levels at the promoter region of *Il-6*, along with its release in the niche of KSCs. As a result, the increased activity of the downstream JAK2/STAT3 signaling pathway in KSCs leads to heightened cell growth and promotion of EMT. This disruption in the cellular microenvironment contributes to a more severe tumor response in the offspring.

Epigenetic modifications at promoter sites have been linked to the excessive production of multiple inflammatory cytokines, with changes in methylation levels being a possible cause. In our study, we focused on prenatal exposure and examined the mRNA levels of specific genes that may regulate the epigenetic landscape of IL-6 in prenatally challenged DFs. We found a significant increase in the levels of TET1 in prenatally exposed DFs. TET1 is responsible for converting 5-mC to 5-hmC, which makes the target region more active by reducing methyl marks. Increased 5-hmC conversion was validated via immunostaining on primary DFs (Fig. 6A). Prenatally exposed DFs showed a higher transition of 5-mC to 5-hmC compared to unexposed cells. The number of cells with positive 5-mC modification decreased in previously exposed DFs with significant rise in 5-hmC marks. We also confirmed an upregulation (1.8 ± 0.06 fold) of TET1 at protein level through western blotting in prenatally exposed DFs, compared to the control group (Fig. 6B). We also observed a significant upregulation of IL-6 mRNA levels by 8.1 ± 1.4 fold in the prenatally exposed cells (Fig. 6C).

Furthermore, we assessed the ratio of 5-hmC to 5-mC in a 2 kb upstream flank of the IL-6 gene promoter to examine any alterations due to disturbed TET1 function (Fig. 6D, E). We performed MeDIP on primary DFs isolated at PND 2 and found an increase of 8.4 ± 1.5% in the 5-hmC/5-mC ratio compared to the input in prenatally exposed DFs, while control DFs showed a decrease in the ratio with 0.5 ± 0.1% (Fig. 6D). Another site showed a similar pattern, though with less prominent modifications, with an increase of 1.6 ± 0.16% in the prenatally exposed group compared to the decreased ratio in control cells of 0.4 ± 0.09% (Fig. 6E). These results confirm an increased rate of conversion in 5-hmC at the *Il-6* promoter, leading to increased gene expression, subsequent high synthesis, and release of IL-6 by DFs. This further contributes to the transformation of the microenvironment and primes KSCs for enhanced skin tumorigenesis.

## Discussion

The current study shows how developmental exposure to environmental pollutants such as As can affect cellular plasticity and epigenetically alter genes that have a key role in creating a tumor microenvironment. In a previous study^18^, we described how prenatal As exposure can reduce tumor latency and accelerate skin tumorigenesis in mice offspring upon challenge with DMBA/TPA (Supplementary Fig. 2A). The enhanced tumor response was related to altered dynamics of transitly-amplifying progenitor cells, i.e., basal keratinocyte cells (BKCs). Being an immediate progeny of the epidermal stem cell population, i.e., KSCs, BKCs are transient possessors of stem-like properties. In the skin, KSCs are typically quiescent or slow dividing until activated by injury or other stimuli^22^. Upon insult, KSCs activate and divide to form clonally expanding BKCs promoting tumorigenesis under a favorable tumor microenvironment ^23^. The current study confirms the role of As -conditioned fibroblasts in creating a tumor microenvironment that activates KSCs and promotes tumorigenesis. Unexposed KSCs were cultured in conditioned media derived from As -exposed fibroblasts (EMEM-CM) to assess the role of secreted factors in modulating KSCs' function. We show how As -conditioned fibroblasts play a key role in the creation of pro-tumorigenic microenvironment in exposed offspring through elevated secretion of IL-6, further increasing the proliferation and migration potential of resident KSCs.

The As -conditioned fibroblast isolated from 2-day-old offspring showed high levels of α-SMA, Collagen IV, and Fibronectin, indicating encouraged fibroblast activation (Fig. 2). Previous studies have shown that AFs, also known as CAFs, can promote therapeutic resistance, cancer development, and progression^24^. It is widely acknowledged that AFs have a clinically significant impact on cancer onset, treatment effectiveness, and patient prognosis in multiple cancer types. We discovered that accelerated carcinogenesis caused by stem cell niche transition was related to increased expression of α-SMA, an AFs marker^25^ in the skins of prenatally exposed offspring at 18 weeks (Fig. 1F) and PND 2 (Fig. 1K). Studying the interactions between cancer cells and AFs in reliable experimental models has been a major challenge. To address this, we successfully maintained primary KSC cultures in DFs-conditioned media, derived from prenatally exposed mice to mimic the tumor microenvironment and examine the effects.

The microenvironment plays a fundamental role in shaping the fate of slow-cycling KSCs by controlling multiple factors, including the inflammatory milieu. To produce a microenvironment that is favorable for tumor development, AFs release a range of molecules (such as IL-6, TGFβ-1, and bFGF) that activate critical signaling pathways (WNT, Notch, Hedgehog and EMT signaling) in stem cells. Previous studies have shown that fibroblasts can stimulate primary keratinocytes to exhibit an EMT-like phenotype^26^. In addition to direct contact between AFs and cancer cells, including stem cells, biochemical crosstalk is an important aspect of tumor formation. AFs help cancer cells migrate by modifying the extracellular matrix. Contrarily, they can directly drag cancer cells through epithelial to mesenchymal transition by altering the production of N- and E-cadherins. These findings are consistent with our results, where increased EMT and proliferation in unexposed KSCs were observed when they were cultivated in an IL-6-rich conditioned medium obtained from DFs (Fig. 3F) of As -exposed offspring.

Previous research has demonstrated the significance of IL-6 in tumor growth and metastasis in several cancer types^27^. Interleukin-6 is an essential element in the cancer microenvironment and may be generated by both cancer cells and their associated stromal equivalents, such as CAFs. Its effects can extend beyond the local cancer environment, as it can leak into other tissues and contribute to the development of a pre-metastatic niche. IL-6 functions in maintaining low-grade differentiation of SCC and promoting EMT^28^. Elevated serum IL-6 concentrations have been detected in OSCC (Oral Squamous Cell Carcinomas), resulting in larger tumor size, nodal metastases, and poorer patient survival^29^. Also, increased IL-6 levels are associated with unfortunate responses towards chemotherapy and radiation. Cancer cells exploit the adaptability of stromal fibroblasts to create a microenvironment that supports tumor growth through various signals. The process of EMT, which is crucial during embryonic development, is used by tumor cells to accelerate invasion and metastasis. According to a study, IL-6 promotes EMT in head and neck tumors by altering Snail expression^30^. The constitutive expression of Twist in breast cancer cells can result from inappropriate production of IL-6 and STAT3 phosphorylation^31^. This, in turn, directly inhibits the transcription of E-cadherin, ultimately promoting the invasion and movement of tumor cells, a crucial step in the process of metastasis. Our study found that DFs releasing IL-6 caused KSCs to lose the epithelial marker E-cadherin and acquire the mesenchymal marker N-cadherin (Fig. 3F), resulting in increased migration in the scratch assay (Fig. 5B). These findings were consistent with in vivo observations at PND 2 and 18 weeks, where EMT was clearly depicted (Fig. 1).

IL-6 has been found to activate the JAK2-STAT3 signaling pathway by binding to the IL-6a chain and GP130 receptors, promoting tumor progression^32^. Although the role of AFs and IL-6 in skin cancer has not been extensively studied, our findings show that prenatally AFs release IL-6, which promotes the phosphorylation of JAK2 and STAT3 in KSCs. Blocking this pathway via SC144 significantly reduced cell migration and EMT. Interleukin-6/STAT3 are key contributors to cancer growth and progression^29^. IL-6 activates STAT3 and drives its translocation to the nucleus, where it interacts with regulatory sites to turn on gene expression. Immunoblot and ICC results in Fig. 4B, E, H respectively demonstrate increased translocation of pSTAT3 to nucleus in KSCs. This resulted in consistent translation of downstream targets responsible for cell proliferation and invasion. Ongoing activation of inflammatory mediators like IL-6 may contribute to tumor growth caused by faulty epigenetic modifications. IL6/GP130/STAT3 signaling promotes malignancy in colon cancer, while in gastric cancer, tumor mesenchymal stromal cells release IL-6 to promote EMT in cancer cells, further spreading the disease^33^.

The progression of cancer involves significant changes in genes and epigenetic modifications, such as DNA methylation and histone modifications that can activate or silence genes. These modifications can also occur in somatic cells, indirectly affecting cancer progression through cellular crosstalk. DNA cytosine methylation is a chemical modification found in mammalian genomes that is associated with various pathological processes, including gene regulation, genomic imprinting, and cancer. 5-methylcytosine (5-mC) is distributed throughout the mammalian genome in a non-random way, effectively controlling DNA accessibility to transcription factors and chromatin regulators, contributing to gene regulation and cellular development ^34^. It is essential to note that TET1 is an enzyme that can quickly hydrolyze 5-mC into 5-hmC, which can then be converted into unmethylated cytosine, increasing the accessibility for regulatory proteins and enzymes. Recent studies have shown that exposure to UVB radiation can lead to decreased TET1 activity and methylation in human and mouse skin tissues and cutaneous cell lines, which can downregulate the TGF-/BMP-SMAD-ID4 signaling pathway in epidermal SCCs^35^. Additionally, it is worth mentioning that TET1 is responsible for maintaining 5-hmC in mouse embryonic stem cells and is activated during the reprogramming of fibroblasts^36^. These studies suggest that TET1 plays a crucial role in regulating the transcription of many genes, including inflammatory genes, through modifications such as 5-mC and 5-hmC.

Gene expression is extensively regulated by DNA methylation at certain CpG dinucleotides in the *Il-6* locus. For instance, alveolar macrophages (AMs) in aged mice show considerably decreased levels of methylation at a single CpG dinucleotide, resulting in greater IL-6 production^16^. Fibroblasts isolated from 2-day-old prenatally exposed offspring were also found to have high global levels of TET1 protein which was associated with increased 5-hmC at *Il-6* promoter. This led to a fibroblast-driven increase in the production of IL-6 in the microenvironment surrounding KSCs, leading to EMT and accelerated tumor development in mice. The effects persisted till 18 weeks suggesting permanent changes in epigenetic regulatory pathways due to As exposure (Fig. 6F). Our study reveals TET1-mediated conversion of 5-mC to 5-hmC at *Il-6* promoter resulting in its excessive production and release by DFs in the stem cell niche (Fig. 6D, E).

Our findings show how early life As exposure modifies stem cell niche through altered fibroblast conditioning, leading to accelerated skin tumorigenesis. It also sheds light on how exposure to environmental toxicants like As can alter the epigenetic landscape, modify the tumor microenvironment, and promote tumor growth.

### Supplementary Information

Supplementary Information.

## Data Availability

The datasets generated and analyzed during the current study are submitted to CSIR-IITR repository and will be made available by corresponding author on request.
