# Supplementary material for: Activated fibroblasts modify keratinocyte stem niche through TET1 and IL-6 to promote their rapid transformation in a mouse model of prenatal arsenic exposure
Source: Sci Rep. 2024 Mar 22;14:6904. doi: 10.1038/s41598-024-56547-8 (PMC10959921; doi:10.1038/s41598-024-56547-8)
Supplement: Supplementary file 1 — Supplementary Information. [file 41598_2024_56547_MOESM1_ESM.pdf]

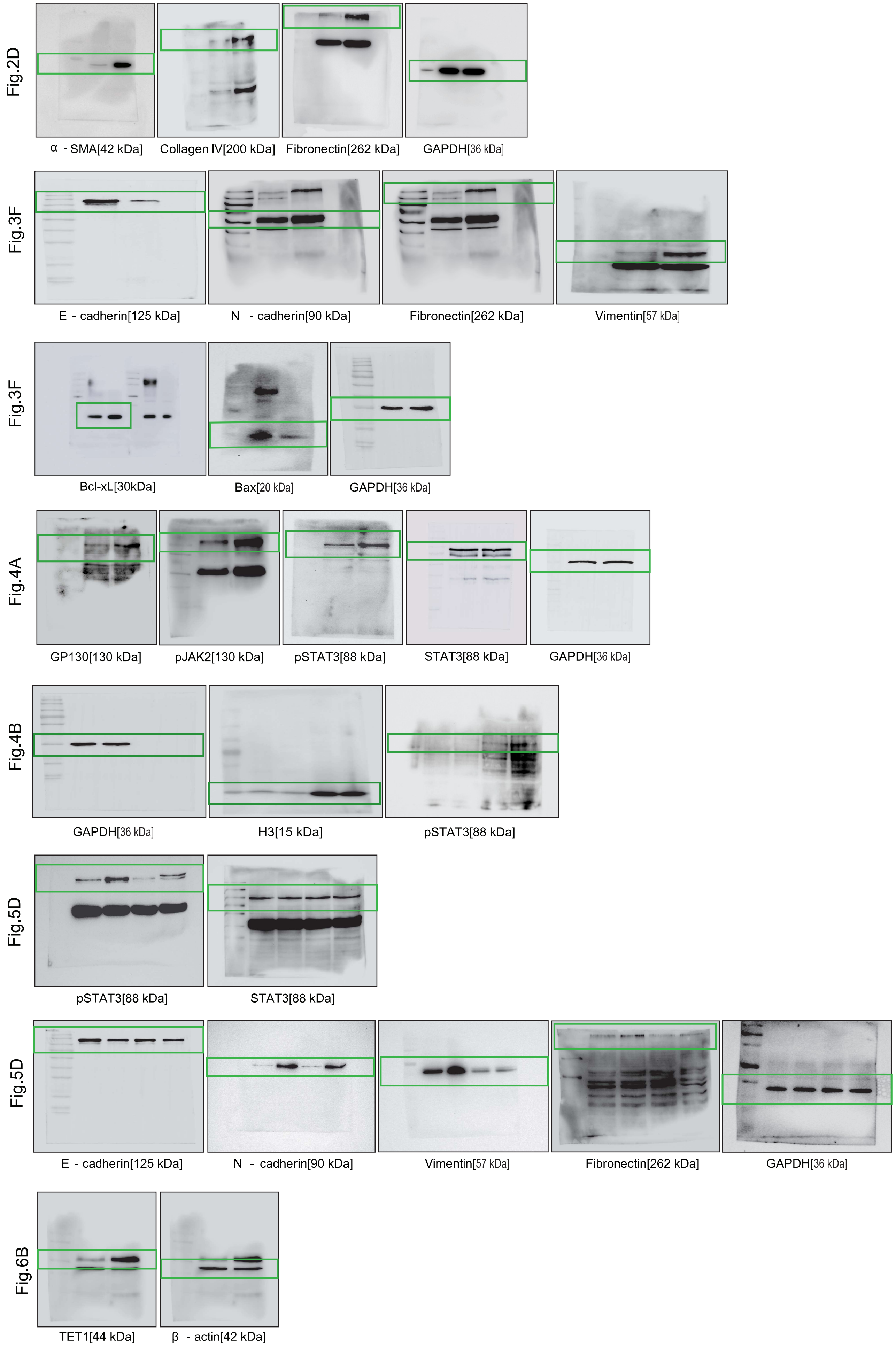

Supplementary Figure 1. Untrimmed blot images corresponding to the respective figures captured via ChemiDocTM MP (BioRad) are shown.

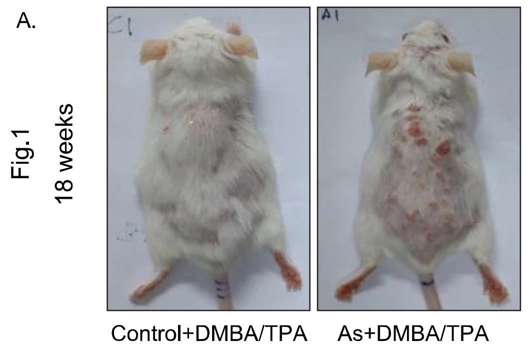

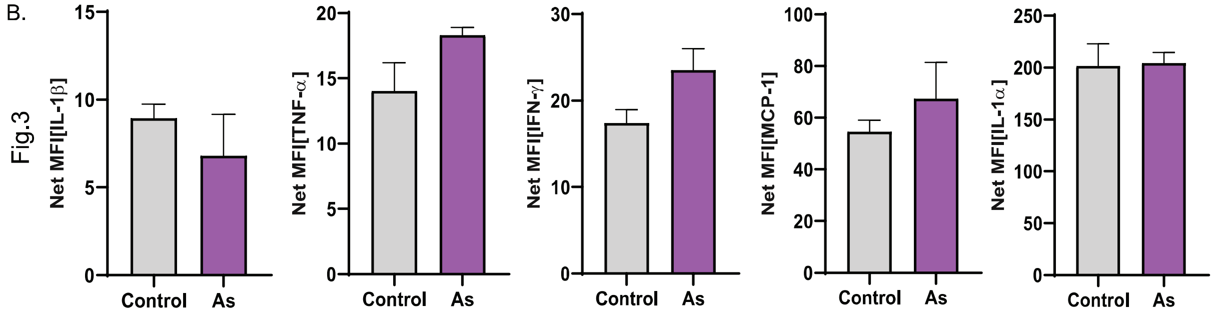

Supplementary Figure 2. (A) Illustrates a higher incidence of tumor formation in offspring exposed prenatally to As, after the administration of DMBA/TPA. (B) The presented graphs display the levels of cytokines examined in the supernatant of cultured DFs.

Supplementary Table 1. List of materials used with source information.

Antibodies	Source	Cat no.
Anti-E cadherin	Abcam	Ab185732
Anti- N cadherin	Invitrogen	Tc25351224
Anti-Fibronectin	Abcam	Ab62635
Anti-Vimentin	Cloud clone	Pab331mu01
Anti-α-SMA	ABclonal	pAb (A7248)
Anti-Collagen IV	Abcam	ab6586
Anti-GP130	Invitrogen	bs- 1459R
Anti-pJAK2	Abcam	ab32101
Anti-pSTAT3	Abcam	ab76315
Anti-STAT3	CST	12640S
GAPDH	CST	2118S
Anti-Rabbit IgG Biotin antibody	Sigma Aldrich	B8895
Anti-*Igf2r*	CST	D8Z3J
Anti-Gαq	CST	D5V1B
Anti-TET1	Santa Cruz	sc-163443
Anti-5-hydroxymethylcytosine (5-hmC)	Abcam	ab106918
Anti-5-methylcytosine (5-mC) antibody	Abcam	D3527

**Reagents**	**Source**	**Cat no.**
Sodium (meta) arsenite	Sigma Aldrich	S7400
7,12-Dimethylbenz [a]anthracene	Sigma Aldrich	D3254
Phorbol 12-myristate 13-acetate	Sigma Aldrich	P8139
Paraformaldehyde	Sigma Aldrich	158127
Sodium citrate(tribasic)	Sigma Aldrich	C8532
Albumin, Bovine, Fraction V	Puregene	PG-2330
Streptavidin Horseradish Peroxidase (HRP)Conjugate	Invitrogen	434323
Hematoxylin	Sigma Aldrich	H3136
3,3'- Diaminobenzidine Enhanced Liquid Substrate System tetrahydro–chloride	Sigma Aldrich	D3939
5'Bromo-2'- deoxyuridine	Sigma Aldrich	B5002
EMEM with EBSS, Non-Essential Amino Acids, & L-Glutamine without Calcium Chloride	Lonza	06-174G
Dulbecco's Phosphate Buffered Saline	Sigma Aldrich	D8537
2.5% Trypsin without EDTA	Gibco	15090046

Chelex 100 sodium form 50-100 mesh	Sigma Aldrich	95577
FBS	Gibco	10437028
Mitomycin C	Sigma Aldrich	M0503
Hydrocortisone	Sigma Aldrich	H0888
Insulin solution	Sigma Aldrich	10516
Cholera toxin	Sigma Aldrich	C9903
Calcium chloride	Sigma Aldrich	C4901
T3	Sigma Aldrich	T5516
Dulbecco's Modified Eagle Medium/ Nutrient Mixture F12, 3:1	Himedia	AT189
TrypLE™ Express	Gibco	12604021
**Kits**	**Source**	**Cat no.**
DeadEnd™ Colorimetric TUNEL System	Promega	G7131
Bio-plex pro mouse cytokine 5-plex assay	Biorad	custom made

Supplementary Table 2. Primers used in mRNA and chip analysis

**Gene**[mRNA analysis]	**Primer sequence**
Tet 1	forward: CTGGGATAGTGCTAGGCTGC<br>reverse: GCAGCTCTGCAAACTGCTTT
Il-6	forward: GTGGTAATTCTGCAATGTGGGG<br>reverse: CAAATGCCCCTGAAAGCACC
**Gene** [ChIP analysis]	**Primer sequence**
Il-6 Set 1	forward: AGCAGTGGGATCAGCACTAA<br>reverse: TCACCCACTTTACCCACCTG
Set 2	forward: TCACCCACTTTACCCACCTG<br>reverse:GGAGAGGGAGTGTGTGTCTT
